# Epigenetics of Animal Personality: DNA Methylation Cannot Explain the Heritability of Exploratory Behavior in a Songbird

**DOI:** 10.1093/icb/icaa138

**Published:** 2020-11-12

**Authors:** Kees van Oers, Bernice Sepers, William Sies, Fleur Gawehns, Koen J F Verhoeven, Veronika N Laine

**Affiliations:** 1 Department of Animal Ecology, Netherlands Institute of Ecology (NIOO-KNAW), Droevendaalsesteeg 10, Wageningen, 6708 PB, The Netherlands; 2 Behavioural Ecology Group, Wageningen University & Research, Wageningen, P.O. Box 338, 6700 AH, the Netherlands; 3 Bioinformatics Unit, Netherlands Institute of Ecology (NIOO-KNAW), Droevendaalsesteeg 10, Wageningen, 6708 PB, The Netherlands; 4 Department of Terrestrial Ecology, Netherlands Institute of Ecology (NIOO-KNAW), Droevendaalsesteeg 10, Wageningen, 6708 PB, The Netherlands

## Abstract

The search for the hereditary mechanisms underlying quantitative traits traditionally focused on the identification of underlying genomic polymorphisms such as single-nucleotide polymorphisms. It has now become clear that epigenetic mechanisms, such as DNA methylation, can consistently alter gene expression over multiple generations. It is unclear, however, if and how DNA methylation can stably be transferred from one generation to the next and can thereby be a component of the heritable variation of a trait. In this study, we explore whether DNA methylation responds to phenotypic selection using whole-genome and genome-wide bisulfite approaches. We assessed differential erythrocyte DNA methylation patterns between extreme personality types in the Great Tit (*Parus major*). For this, we used individuals from a four-generation artificial bi-directional selection experiment and siblings from eight F2 inter-cross families. We find no differentially methylated sites when comparing the selected personality lines, providing no evidence for the so-called epialleles associated with exploratory behavior. Using a pair-wise sibling design in the F2 intercrosses, we show that the genome-wide DNA methylation profiles of individuals are mainly explained by family structure, indicating that the majority of variation in DNA methylation in CpG sites between individuals can be explained by genetic differences. Although we found some candidates explaining behavioral differences between F2 siblings, we could not confirm this with a whole-genome approach, thereby confirming the absence of epialleles in these F2 intercrosses. We conclude that while epigenetic variation may underlie phenotypic variation in behavioral traits, we were not able to find evidence that DNA methylation can explain heritable variation in personality traits in Great Tits.

## Introduction

Most quantitative traits, such as behavioral traits, consist of both a considerable nonheritable environmental component, and a part that is attributed to heritable or so-called genetic effects (Benzer 1973; Kearsey and Pooni 1996; Barton and Keightley 2002; Stirling et al. 2002). The search for the molecular mechanisms underlying the heritable component of these traits traditionally focused on the identification of associations with underlying genomic polymorphisms such as single-nucleotide polymorphisms (SNPs) or other structural variants (Sokolowski 2001; Boake et al. 2002). Although many candidate genes were identified with often small effects, most of these studies were able to explain just a fraction of the heritable variation in behavioral traits and the hereditary mechanisms still largely remain unknown (Bell and Dochtermann 2015; Laine and van Oers 2017).

Exploratory behavior is a widely-used behavioral trait (Hughes 1997) to measure the behavioral response to a novel environment (NE), often studied in the context of animal personality (Réale et al. 2007). Exploratory behavior is known to have a moderately heritable component of on average 58% (van Oers and Sinn 2013), but at the same time, it is affected by early developmental factors (Carere et al. 2005; Krause and Naguib 2011; van Oers et al. 2015). Large effort has been made to unravel the structural genomic variation underlying the additive and nonadditive genetic component reflecting the heritable component of exploratory behavior (van Oers and Mueller 2010). Some associations of low effect size with several SNPs within candidate genes have been reported (Fidler et al. 2007; Timm et al. 2019; but see e.g., Edwards et al. 2015), and genome-wide association studies and quantitative trait locus studies have revealed some regions of interest in the genome (Santure et al. 2015; Kim et al. 2018), albeit without showing any clear and consistent pattern (Crusio et al. 2013; Laine and van Oers 2017). In conclusion, the genomic structure underlying heritable variation in exploratory behavior remains poorly understood.

Several reviews have suggested that apart from structural variation in genes, also epigenetic mechanisms may help us to fill this gap in our knowledge on the inheritance of behavioral traits (Stamps and Groothuis 2010; Groothuis and Trillmich 2011; Trillmich and Hudson 2011). Epigenetic mechanisms can be defined as those biochemical mechanisms that stably alter gene expression by affecting either transcription or translation without a single change in the primary nucleotide sequence of the genome (Richards 2006). In many organisms, epigenetic mechanisms alter gene expression as a response to a variety of internal and external factors (Law and Jacobsen 2010). For example, DNA methylation levels of cytosines in the context of CpG dinucleotides, which are CG dinucleotides (5’-cytosine guanine-3’) separated by a phosphate (p) group, one of the best studied epigenetic mechanisms, have been shown to change during the course of development under the influence of DNA methyltransferases (Hashimshony et al. 2003). These changes are directly affecting the activity of specific genes that are suppressed or repressed during different developmental stages. CpG methylation has not only been found to predictably vary over developmental stage, but may also change due to environmental factors. The environment can, on one hand, cause methylation changes during development under influence of, for example nutrition, early-life stress and stochastic processes during two so-called sensitive periods, when after a global demethylation methylation is reestablished (Faulk and Dolinoy 2011). Typically, these changes are often long-lasting throughout an individual’s lifetime (Faulk and Dolinoy 2011). On the other hand, changes induced rhythmic through seasonally varying factors (Stevenson and Prendergast 2013; Viitaniemi et al. 2019), where predictable changes in the environment may cause repeatable DNA methylation fluctuations throughout a lifetime. However, such persistent changes in DNA methylation are most likely caused by epigenetic inheritance in the broad sense, which is the inheritance of developmental variation throughout mitotic cell-division within an organism (Jablonka and Raz 2009).

Much less is known on whether between-individual variation in DNA methylation can be a target for natural selection itself (Ledon-Rettig et al. 2012; Verhoeven et al. 2016; Hu and Barrett 2017). More specifically, the hypothesis that DNA methylation can be stably inherited via the germline to next generations (inheritance in the narrow sence) would allow for DNA methylation to be selected on and hence impact evolutionary processes (Heard and Martienssen 2014; Bošković and Rando 2018). Examples of the existence of such eppi-alleles are mainly found in plants (Heard and Martienssen 2014) and in wild vertebrates, few studies have provided only indirect evidence that DNA methylation might be under natural selection. In Darwin’s Finches, DNA methylation has been shown to accumulate during speciation, while the phylogenetic distance was unrelated to genetic variation in the form of the number of copy number variant mutations (Skinner et al. 2014). In Great Tits (*Parus major*), genes in genomic regions with signals of positive selection, so-called selective sweep regions, were found to have higher levels of CpG methylation and lower non-CpG methylation compared to genes that were outside of these selective sweep regions (Laine et al. 2016). This points to the possibility that methylation variation is affected by past selection. However, an alternative explanation is that epigenetic variation only facilitates genetic selection or that selection is acting on genetic variants that in their case affect methylation (Verhoeven et al. 2016).

In order to test if a part of the heritable variation in a quantitative behavioral trait, such as exploratory behavior, could be explained by the transgenerational inheritance of stable epigenetic variation, we here investigated the correlated response of erythrocyte DNA methylation to artificial phenotypic selection on early exploratory behavior (EEB), a validated personality trait in a European songbird, the Great Tit (*P. major*). DNA methylation in the Great Tit genome is known to show a typical pattern known for vertebrates (Laine et al. 2016). Methylation levels are relatively high in intergenic regions, with percentages reaching around 50. Close to transcription start sites (TSSs) of genes, the methylation levels in CpGs drop, to rise again after the TSS, where gene body (introns and exons) percentages reach comparable levels to intergenic regions. This hypomethylation around the TSS is associated with the presence of so-called CpG-islands (CGIs), CG-rich areas with promotor regions of genes. We have shown before that in Great Tits, levels of DNA methylation in red blood cells, as we used here, are highly correlated (∼0.8) to methylation levels in brain (Derks et al. 2016).

We assessed whole-genome differential methylation patterns between pools of individuals from a four-generation artificial bi-directional selection experiment and between siblings with extreme personality phenotypes originating from eight F2 intercross families using a combination of reduced representation bisulfite sequencing (RRBS) and whole-genome bisulfite sequencing (WGBS) methods, validated for *P. major* (Derks et al. 2016; Laine et al. 2016; Mäkinen et al. 2019). We identified differential CpG methylation in several CpGs in one candidate gene, *DRD4* in a former study using pyrosequencing, suggesting that epigenetic expression regulation is partly heritable (Verhulst et al. 2016). We, therefore, predicted that due to selection on exploratory behavior, founder effects and drift, genetic differences may have accumulated in DNA methylation between the two lines. By using the intra-family comparison between extreme phenotypes in the F2 cross, we expect to only detect those differentially methylated CpGs (DMC) that are due to artificial selection, which is expected to be a subset of the differences between the lines. By using RRBS on a subset of CpG sites on an individual level, we were able to focus on individual level differences of fewer, but functionally relevant CpGs.

We find no differentially methylated sites when comparing the selected personality lines, providing no evidence for so-called epialleles. Using a pair-wise individual sibling design in the F2 intercrosses, we show that genome-wide DNA methylation profiles of individuals are mainly explained by family structure, indicating that the majority of variation in DNA methylation in CpG sites between individuals can be explained by genetic differences or less likely, due to stable transgenerationally inherited methylation patterns. Although we found some candidate CpGs explaining behavioral differences between siblings in these F2 intercrosses, we conclude that while epigenetic variation may be heritable and underly phenotypic variation in behavioral traits, we did not find evidence that selection on exploratory behavior had consequences for co-selection on DNA methylation. Therefore, we conclude here that variation in DNA methylation cannot explain heritable variation in personality traits in Great Tits.

## Material and methods

### Study population

The Great Tit (*P. major*) is a very common passerine bird species, inhabiting all types of wooded areas throughout Europa, Asia, and parts of Northern Africa. Birds used in this study originated from an artificial selection experiment on exploratory behavior (Drent et al. 2003; van Oers et al. 2014). In brief, for the parental generation, nestlings of 10 days old were collected from natural nests and transferred to the aviary facilities of the Netherlands Institute of Ecology (NIOO-KNAW), The Netherlands. These nestlings were hand raised until independence and tested for a combination of two behavioral tests between 1 and 2 months after hatching. A NE test, where a bird was allowed to explore a novel room of 2 × 4 × 2.5 m in which five artificial trees were placed and two novel object (NO) tests, in which a Pink Panther model and a penlight battery were introduced in the home cage of the bird. The NE test resulted in a score between 0 and 10, where a score of 10 was given to birds that visited four out of five trees within 1 min after entering the room and a score of 0 if they did not >3 trees within 10 min. For each NO test, a bird could gain a maximum of five when the bird pecked the NO, resulting in a maximum score of 10 for the two NO tests together. This sums-up to a score between 0 and 20 for EEB. Birds from the parental generation were selected for fast (high scores; fast exploring [FE]) or slow (low scores; slow exploring [SE]) EEB by pairing up males and females with either high or low scores, taking into account family relationships. They were housed in pairs in half-open aviaries to produce first-generation (F1) eggs. To minimize pre- and post-hatching non-genetic maternal effects, eggs were transferred to a nest in a natural population, where they hatched. To match rearing conditions for the two selection lines (SELs), chicks were cross-fostered one day after hatching, in such a way that half of a slow brood were raised together with half of a fast brood. Foster nests were chosen based on their timing of incubation without taking parental characteristics into account. Ten days after hatching, the nestlings were then brought to the NIOO-KNAW facilities for standardized hand rearing. This procedure was repeated for four generations (F4) resulting in bidirectional SELs (see Drent et al. [2003] for details). Males and females from the F4 were subsequently crossed by pairing them with partners from the other SEL producing reciprocal F1-intercross (F1C) offspring using the same procedure as described above, where all F1C individuals are expected to be heterozygous at relevant loci (Lynch and Walsh 1998). Twenty F1C Females were then randomly chosen to produce an F2-intercross generation (F2C; see van Oers et al. 2014 for details), where all the relevant loci are expected to segregate out. EEB has a realized heritability of 54%, calculated from this selection experiment (Drent et al. 2003). Heritability estimates of exploratory score range from 0.22 to 0.61 in our wild population (Dingemanse et al. 2002).

We used two different genetic groups of birds for this study to create three datasets. As first genetic group for the first dataset, we used six unrelated FE and six unrelated SE individuals originating from the F4 of the EEB SELs. For both SE and FE, we pooled DNA of three males and three females to produce an SE (SE-SEL) and an FE pool (FE-SEL), respectively, for WGBS. The second genetic group, F2C, was used for two datasets. From eight F2C families, we chose the most extreme FE bird (${\overset{-}{x}}_{\mathrm{EEB}}$ = 15; range 14–17; FE-IND) and the most extreme SE bird (${\overset{-}{x}}_{\mathrm{EEB}}$ = 1; range 0–5; SE-IND) per family for RRBS sequencing using 16 individual libraries (see below). As a second dataset for this genetic group, we randomly selected six out of these 8 F2C families (12 individuals) for creating DNA pools to produce an FE-F2C and an SE-F2C pool for WGBS (see below), each consisting of six F2C individuals. For this, small blood samples (10 µL) that were previously collected by puncturing the jugular vein were used that were stored in 1 mL Cell Lysis Buffer (Gentra Puregene Kit, Qiagen, USA) until analysis. DNA was extracted from 250 µL whole blood solution, using a Gentra Puregene Kit (Qiagen, USA) following the manufacturer’s protocol and concentration was determined using a Nanodrop 2000 (ThermoThermo Scientific, USA). DNA was stored in DNA Hydratation Solution (Qiagen, USA). By running 1.5 µL on a 1% agarose gel together with a size ladder, the integrity of the DNA was verified.

### WGBS

#### Bisulfite sequencing library preparation and sequencing

We conducted WGBS on pools of both SEL (FE-SEL and SE-SEL) and F2C-FAM (FE-F2C and SE-F2C). For WGBS, detailed sequencing methods are described in Derks et al*.* (2016). In brief, DNA of the four pools of samples (FE-SEL, SE-SEL, FE-F2C, and SE-F2C) was sheared using a Covaris E210 device to ∼700 bp peak fragment sizes. One microgram of sheared and purified DNA was used together with 1 ng sheared Lambda DNA according to the Illumina TruSeq LT DNA sample preparation guide. After this, adapter ligated DNA was purified using AmpureXP beads (Agencourt) and bisulfite converted using the EpiTect Plus Bisulfite Kit (Qiagen). Converted DNA was purified and split over three parallel reactions using Pfu Cx hotstart DNA polymerase (Agilent Technologies) and 18 PCR cycles. PCR products were pooled and final libraries were quantified using a Bio analyzer DNA 1000 chip (Agilent Technologies). WGBS libraries were sequenced on an Illumina HiSeq2500 at Wageningen University & Research Next-Generation Sequencing facilities. Phix, together with Illumina adapter sequences, were removed after sequencing by the sequencing facility. FE-SEL, SE-SEL, FE-F2C, and SE-F2C libraries generated 145 M (36 Gb), 201 M (50 Gb), 203 M (50 Gb), and 192 M (48 Gb) paired-end (125 bp) reads, respectively, corresponding to an average coverage of 6.6×, 13.9×, 14.9×, and 17.9×, respectively, for CpG sites in the four WGBS data sets (Supplementary Table S1).

#### Quality control, trimming, and filtering of raw reads

Sequencing quality of WGBS read libraries was inspected using FastQC version 0.11.8 (Andrews 2010). FastQ screen version 0.11.1 (Wingett and Andrews 2018) in bisulfite mode was used to detect possible contaminations caused by sampling and sequencing, to check read coverage, ATCG content, Ncontent, and adapter content. Premade index databases were used to check for contamination, including vectors (UniVec Core), FastQC adapters (Andrews 2010), Phix (*Coliphage* phi-X174, complete genome), *Escherichia coli* (*E. coli str. K-12 substr. MG1655*, complete genome), *Homo sapiens* (Genome Reference Consortium Human Build 38), and *Arabidopsis thaliana* (*A. thaliana* (thale cress), TAIR10). A *P. major* index was created using the reference genome version 1.1 (NCBI Assembly GCA_001522545.3; Laine et al. 2016) to confirm presence of *P. major* sequences. Contents of contamination levels lower than 3% were considered as absent. Multiqc version 1.7 (Ewels et al. 2016) was used to summarize the results of FastQC and FastQ screen.

Trimming and filtering were performed using Trimmomatic version 0.39 (Bolger et al. 2014). Read quality was improved by removing the first five base pairs (bp) at the start of the reads, since bisulfite conversion is less efficient there. Reads were filtered and trimmed for a minimum base quality of 20 Phred at the start and end of a read, a sliding window of 4 bp with average quality > 20 Phred, an overall average quality > 20 Phred and at last minimum read length of 40 bp. For adapter clipping, Trimmomatic was used with the adapter database (Data from March 30, 2017; Andrews 2010) using settings: Illuminaclip: FILE : 2:30:10. Efficiency of read trimming and filtering was confirmed by FastQC, FastQ screen and Multiqc for all WGBS libraries (Supplementary Fig. S1).

#### Alignment, deduplication, and methylation calling

Reads were aligned to the reference genome using BS-Seeker2 version 2.1.8 (Guo et al. 2013) using the aligner Bowtie2 version 2.3.5 and gcc version 7.3.0 (Langmead and Salzberg 2012). An index of the *P. major* reference genome version 1.1 (Laine et al. 2016) was created using bs_seeker2-build.py.

WGBS datasets suffer from duplication trough PCR duplicates. We conducted deduplication from PCR duplicates using samtools 1.9 (htslib 1.9). BAM files were name sorted (*sort -n*), mate fixed (*fixmate -r -m*), position sorted (*sort*), and duplication marked and removed (*markdup -r*).

Methylation calling was done using CGmapTools (Guo et al. 2018) on the bam file, which was sorted with samtools *sort*. As settings we used convert bam2cgmap, *P. major* reference genome 1.1 and the rmOverlap setting to remove possible overlap due to paired end sequencing.

#### Filtering, differential methylation analyses, and visualization

Differentially methylated site analysis was performed in multiple steps using R version 3.6.1 (R Core Team 2018). We filtered for CpG sites using the *CGmap* function of CgmapTools. CpG sites were filtered for 10× minimum coverage and if a site was completely unmethylated or methylated (0% or 100%) for all pools, the site was removed. After inspection of plots between coverage and significance, we detected a strong association between coverage and differential methylation: sites with high coverage showed a larger chance of having significant differential methylation. We considered this a technical artefact and to avoid this coverage issue, we ranked the sites according to their coverage and the 99.9% sites with the lowest coverage were retained. The final datasets consisted of only those CpG sites that were present after filtering in both the FE-pool and SE-pool in the SEL (1,521,921 CpGs) as well as in the F2C (4,963,579 CpGs) comparison.

The significance of differential methylation for each of the individual C in a CpG site for the WGBS data sets (SE-SEL, FE-SEL, SE-F2C, and FE-F2C) was calculated using a Generalized Linear Model. As a dependent variable, we used the *cbind* function to create a response variable with C’s (methylated Cs; successes) and Ts (unmethylated Cs; failures) combined, and we included Line (SE or FE) as fixed variable using a binomial error structure and a logit link function.

Sites with singularity or with converge warnings were omitted. Throughout we conducted a multiple-testing correction using genome-wide Bonferroni thresholds: ^10^log(0.05/1,521,921) = 7.48 for the FE-SEL vs. SE-SEL comparison and ^10^log(0.05/6.5M) = 8.00 for the FE-F2C vs. SE-F2C comparison, and a minimum methylation ratio difference of 10% was used as threshold.

### RRBS

#### Bisulfite sequencing library preparation and sequencing

We conducted RRBS on all 16 individuals originating from 8 F2C families individually (FE-IND and SE-IND). For this, high-quality DNA (1 µg) of the 16 F2C individuals was used by the Roy J. Carver Biotechnology Center (University of Illinois, Urbana, USA) for generation of RRBS libraries following standard protocols. Using the restriction enzyme MSpI, DNA was digested, and the resulting fragments were size selected to a range between 20 and 200 bp using manual cutting after agarose gel electrophoresis. Size selected DNA was column-purified after it was bisulfite-converted using the EpiTect Bisulfite Kit (Qiagen). Libraries were quantified using Qubit (Life Technologies, USA) and size-analyzed using am Agilent Bioanalyzer DNA7500 DNA chip (Agilent Technologies, USA) and diluted to 10 nM. To ensure high accuracy quantification for consistent pooling of barcoded libraries and maximization of the number of clusters in the Illumina flow cell, dilution was further quantitated by qPCR on an ABI 1900. The 16 libraries were sequenced single-end (100 bp) on 2 Illumina HiSeq2500 lanes using a HiSeqSBS sequencing kit version 4 in such a way that the families and SE and FE were balanced over lanes to avoid lane effects, yielding on average 18 M reads per individual (Supplementary Table S1). Reads were demultiplexed after sequencing using bcl2fastq version 2.17.1.14 (Illumina). RRBS libraries were quality-checked by the Roy J. Carver Biotechnology Center and they trimmed adapters from the raw reads. No further trimming or filtering was needed.

#### Alignment of reads to reference genome and methylation calling

Reads were aligned to reference genome using BS-Seeker2 version 2.1.8 (Guo et al. 2013) using the aligner Bowtie2 version 2.3.5 and gcc version 7.3.0 (Langmead and Salzberg 2012). An RRBS index of the *P. major* reference genome version 1.1 (Laine et al. 2016) was created using bs_seeker2-build.py.

Methylation calling was done using CGmapTools (Guo et al. 2018) on the bam file, which was sorted with samtools *sort* with convert bam2cgmap using the *P. major* reference genome 1.1.

#### Filtering, differential methylation analyses

Differentially methylated site analysis was performed in multiple steps using R version 3.6.1 (R Core Team 2018). We filtered for CG sites using the *CGmap* function of CGmapTools. CpG sites were filtered for 10x minimum coverage and if a site was completely unmethylated or methylated (0% or 100%) for all individuals, the site was removed. To avoid coverage issues with the analysis, the sites were ranked according to their coverage and the 99.9% sites with the lowest coverage were retained. Only those sites that were present in at least 7 families and that were present in at least 14 out 16 individuals were used for the differential methylation analysis (233,198 CpGs).

Differential methylation for each C in a CpG site was calculated using Generalized Linear Mixed Model (GLMM) with lme4 version 1.1-21 (Bates et al. 2015) with cbind (C, T) as dependent variable, and personality (FE or SE) as fixed variable using a binomial error structure and a logit function. Family was included as a random factor to account for the fact that SE and FE consisted of two matched birds from the same family. Modeling was done multithreaded using *mclapply*.

A dispersion parameter (λ) was calculated for RRBS using the residuals of the individual GLMMs as the ratio of the variance (sum of residuals) divided by two times the number of fixed and random effects. We removed sites that fell outside of the 95% Highest Posterior Density interval (Supplementary Fig. S5). Sites with singularity or with converge warnings were omitted. Throughout we conducted a multiple-testing correction using a genomewide Bonferroni threshold of −^10^log(0.05/233,198) = 6.67 and a minimum methylation ratio difference of 10% was used as threshold.

### Visualization and downstream analysis

The dendrogram of the F2C individuals with the *ward.d* cluster method and *pearson* distance and the Principal Component Analysis (PCA) plot were made using *Methylkit* version 1.15.3 (Akalin et al. 2012). Plots of coverage against significance and overdispersion correction were generated using *ggplot2* version 3.2.1 (Wickham 2016). Genomewide manhattan plots and QQ plots of differentially methylated sites were generated using *qqman* version 0.1.4 (Turner 2018). Volcano plots were made using the *plot* function.

Significant sites were linked with genomic features using *rtracklayer* version 1.44.2 (Lawrence et al. 2009) and *GenomicFeatures* version 1.36.4 (Lawrence et al. 2013) making use of the *P. major* reference genome 1.1 annotation ID 102. Features used in the analysis were defined as promoter region (2 K upstream of the gene start and 200 in length), TSS region (300 upstream of the TSS and 50 in length), Genes, Upstream (region 10 K bp from the transcription termination site) and Downstream (region 10 K bp downstream of the TSS). Gene information related to the differentially methylated sites was retrieved from NCBI Assembly, Genome and Gene (Kitts et al. 2016). Gene information from ensemble.org and European Molecular Biology Laboratory (EMBL) *P. major* (Madeira et al. 2019) provided insight in functionality of genes and associated proteins.

### Ethics

Bird breeding and animal experiments were approved by an ethical committee (DEC-KNAW licence no. NIOO 14.12 and NIOO 07.03 to K.V.O.).

## Data accessibility

The raw sequencing data underlying this article are available in the National Center for Biotechnology Information (NCBI) SRA repository under BioProject PRJNA208335 (https://www.ncbi.nlm.nih.gov/sra/PRJNA208335). The individual RRBS libraries of the eight fast F2 cross birds are available in the SRA repository under SRX9131258, SRX9131259, SRX9131260, SRX9131264, SRX9131265, SRX9131270, SRX9131271, SRX1634690 (FE-IND), six of which SRX9131258, SRX9131259, SRX9131260, SRX9131265, SRX9131270, SRX9131271 were used for the FE-F2C WGBS pool (SRA repository SRX9157550). The individual RRBS libraries of the eight slow F2 cross birds (SE-IND) are available in te SRA repository under SRX9131261, SRX9131262, SRX9131263, SRX9131266, SRX9131267, SRX9131268, SRX9131269, SRX1634950, six of which SRX9131261, SRX9131263, SRX9131266, SRX9131268, SRX9131269, SRX1634950 were used for the SE-F2C WGBS pool (SRA repository SRX9157551). The FE-EEB WGBS and SE-EEB WGBS pools are available under SRX9157552 and SRX9157553, respectively.

## Results

### WGBS

We performed WGBS in pooled DNA samples derived from whole blood of birds originating from fast (FE-SEL) and slow selection lines (SE-SEL) on exploratory behavior, and in pooled samples of extreme phenotypes originating from their F2 intercrosses (FE-F2C and SE-F2C). Average mappability to the Great Tit reference genome 1.1 of the four WGBS libraries was 59.74% (Supplementary Table S1). After filtering for 10× coverage and percentile filtering a total of 2.02 million CpGs (13.6% of the total number of CpGs in the genome) were covered for the FE-SEL vs SE-SEL comparison and 6.45 million (43.5%) for the FE-F2C vs. SE-F2C comparison (Supplementary Table S1).

**Fig. 1:**
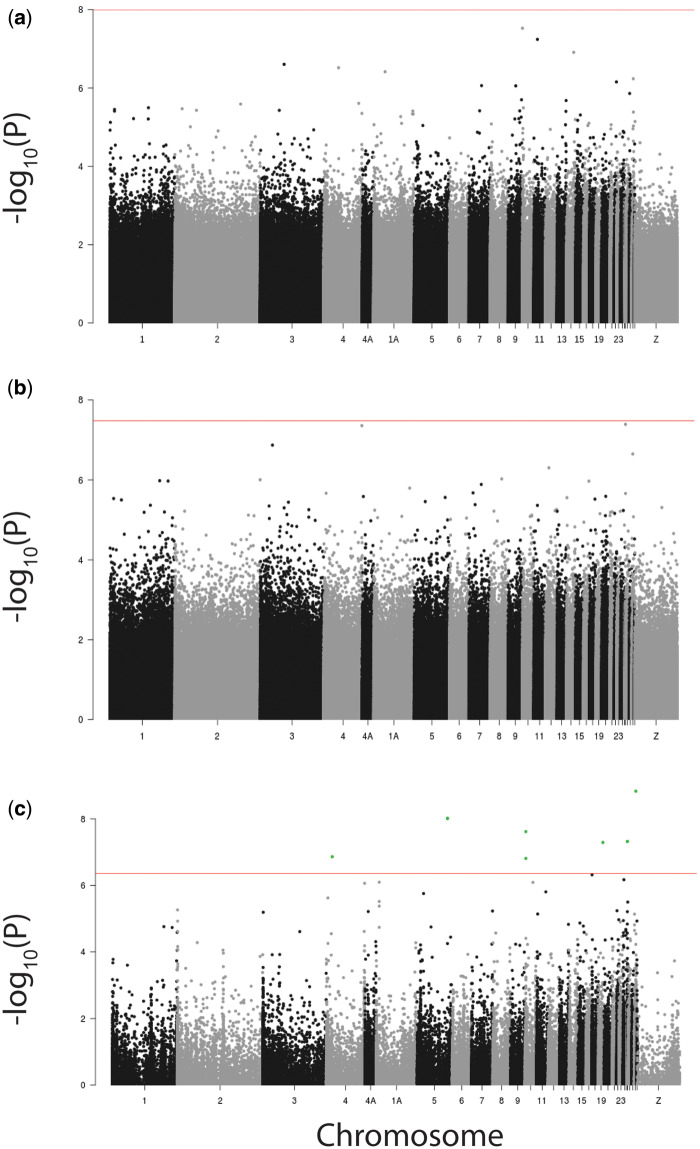
Manhattan plots visualizing the significance of differential methylation of individual CpG sites against the physical *P. major* genome position per chromosome. −log_10_(P) values were calculated from a generalized mixed model with the number of methylated C’s over the number of unmethylated C’s (binary logistic) as dependent variable for the difference between pools of birds originating from (**A**) the fourth generation of selection for fast and slow exploratory behavior (FE-SEL and SE-SEL), (**B**) six extreme slow (SE-F2C) and fast (FE-F2C) phenotypes from F2 intercross families between these lines, and (**C**) from a binary logistic generalized linear model calculating differential methylation between 16 F2 intercross individuals with extreme phenotypes from eight families. Critical Bonferroni corrected −log_10_(p) values are indicated by a horizontal line and genome-wide significant CpG sites are depicted in green.

**Fig. 2:**
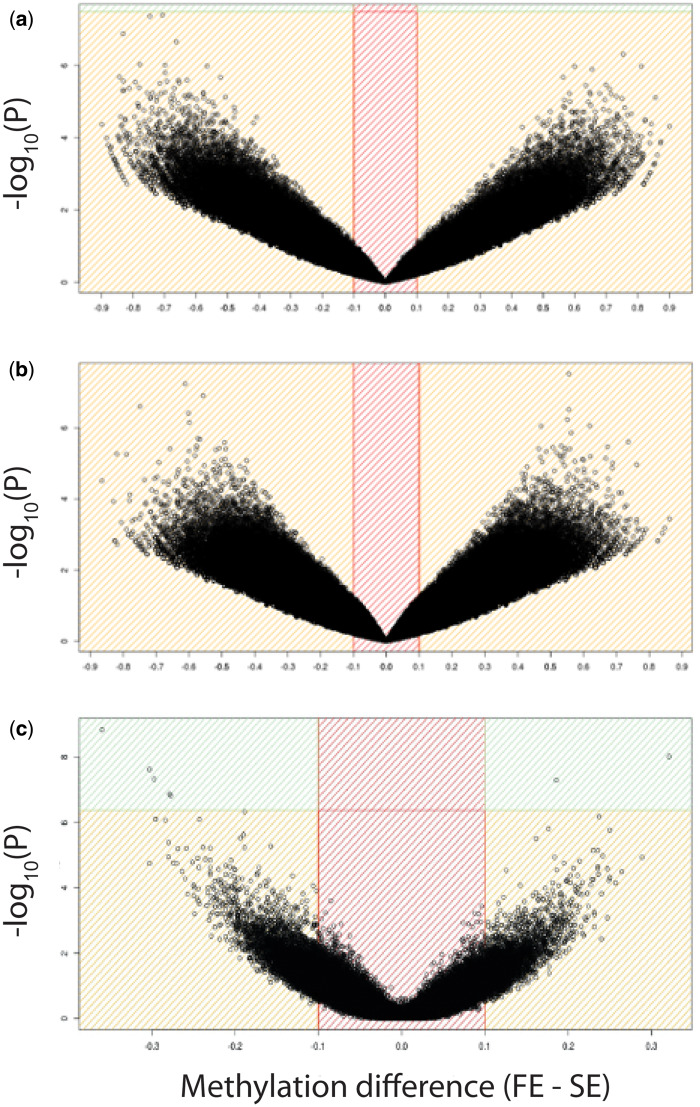
Volcano plots visualizing the difference in DNA methylation between the WGBS pools against the significance derived from the differential methylation analysis for (**A**) pools of five individuals originating from the fourth generation of selection for fast and slow exploratory behavior (FE-SEL and SE-SEL), (**B**) pools of six extreme slow (SE-F2C) and six fast (FE-F2C) phenotypes from six F2 intercross families between these lines, and (C) from a binary logistic generalized linear model calculating differential methylation between 16 F2 intercross individuals with extreme phenotypes from eight families. Methylation differences per site were calculated by subtracting the methylation ratio for SE from site ratios from the FE samples per site. Positive values, therefore, indicate higher methylation levels for FE samples. In the F2C-IND dataset the difference per site was calculated from the mean methylation levels over all samples, subtracting the methylation ratio for SE from site ratios from the FE samples per site. Positive values therefore indicate higher methylation levels for FE samples.

When assessing sites that were differentially methylated among the pools, we found that both for the comparison between the fast and SEL (FE-SEL and SE-SEL) and for the within-family comparison between extreme F2C phenotypes (FE-F2C and SE-F2C) no CpG was significantly differentially methylated after Bonferroni correction (Figs. 1 and 2), indicating an absence of a response to selection on DNA methylation. When combining the two datasets to explore the correlation between significance values of the differential methylation analyses of 1,382,302 CpGs that were present in the SEL and the F2C datasets, we found one CpG that approached significance in both analyses (Supplementary Fig. S7a). One site has borderline significance in both the differential methylation analyses of SEL (−log10(p)  = 5.36 and the differential methylation analyses of F2C (−log10(p)  = 7.24). It is situated in the gene body of RNA polymerase II subunit C (*POLR2C*).

### RRBS

We performed RRBS on individual DNA samples of birds originating from the fastest (FE-IND) and slowest (SE-IND) bird within eight families originating from the F2 intercross. On average, 650,489 (range 509,029–735,704) CpG sites were covered after filtering (Supplementary Table S2).

The average methylation percentage for the 16 RRBS samples was 15.97 (range 14.38–16.93). When clustering the F2C individuals based on their similarity in overall methylation profile, we found that they strongly clustered based on family (Supplementary Fig. S2), suggesting that most variation in their genome-wide methylation profile has a genetically inherited basis. There was no general clustering based on exploratory phenotype, and F2C-FE and F2C-SE birds did not differ in their average methylation percentage (mean ± SEM; F2C-FE: 15.95 ± 0.29; F2C-SE: 15.99 ± 0.33; F_1,14_ = 0.001; P = 0.94) . A PCA analysis did not show any obvious clustering for either family or exploratory type (Supplementary Fig. S3).

When comparing extreme fast F2C individuals with extreme slow F2C individuals, we found seven DMC with −log_10_(p)  > 6.67 and the difference in methylation between FE and SE > 0.10 (Table 1). Five of these CpGs had higher methylation levels in the SE-F2C birds and two showed higher methylation levels in FE-F2C. Six out of these seven sites were situated in annotated genes, of which three were situated in gene bodies. One CpG on chromosome 4 was situated in an intron of LPS-responsive beige-like anchor (*LRBA*) protein and two CpGs were situated 17 bp apart in the last exon of semaphorin 7 A (John Milton Hagen blood group; *SEMA7A*) on chromosome 10. Three significant DMC were situated in the promotor regions of genes. One CpG was found in the TSS of Mitochondrial rRNA methyltransferase 1 (*MRM1*) on chromosome 19, one in the promotor of LOC107214669 on chromosome 25LG2, identified as the scale keratin-like gene at OrthoDB.org v10.1, a bird- and reptile-specific gene and one in the promotor of strawberry notch homolog 2 (*SBNO2*). There was no correlation between significance values of the differential methylation analyses of 80,300 CpGs that were present in the RRBS and the WGBS F2C data sets (Supplementary Fig. S7b).

**Table 1:** The CpG sites that are differentially methylated between eight fast individuals and eight slow individuals from eight F2C families

Chr	Chr Genbank	Position	P-value	−log_10_(P)	Meth.diff (%)	Feature	Gene
4	NC_031771.1	10,648,394	1.37E-07	6.86	−27.88	Gene body	*LRBA*
5	NC_031774.1	54,093,387	9.74E-09	8.01	32.13	–	–
10	NC_031779.1	1,734,466	2.43E-08	7.62	−30.29	Gene body	*SEMA7A*
10	NC_031779.1	1,734,483	1.54E-07	6.81	−27.71	Gene body	*SEMA7A*
19	NC_031787.1	8,103,120	5.14E-08	7.29	18.57	*TSS*	*MRM1*
25LG2	NC_031794.1	166.118	4.79E-08	7.92	−29.71	Promotor	*LOC107214669*
28	NC_031797.1	3,222,562	1.48E-09	8.83	−36.00	Promotor	*SBNO2*

Methylation difference is calculated as FE–SE. Positive values, therefore, indicate hypermethylation in fast individuals.

## Discussion

Here, we have used whole-genome DNA methylation profiling in blood tissue of lines selected for four generations on fast or slow exploratory behavior and their F2 intercross and did not find evidence for the presence of correlated selection on CpG methylation marks. We did not find any differentially methylated site between WGBS pools of six unrelated birds from the fast and slow lines, nor did we find any CpG to be differentially methylated when comparing WGBS pools of two family members with extreme exploratory phenotypes of the F2 intercross between the fast and the slow line using WGBS. When conducting a differential methylation analysis on a reduced set of CpGs on an individual level using RRBS, we did find some interesting candidate CpGs. These candidates did not overlap with the whole-genome WGBS analysis.

This result adds to the studies that fail to find evidence for transgenerational epigenetic inheritance of the so-called epialleles in birds (Guerrero-Bosagna et al. 2018) that in part can likely be explained by the lack of genomic imprinting in birds (Frésard et al. 2014; Zhuo et al. 2017). In fact, in general, these stable CpGs with a Mendelian inheritance are mainly known from plants and some invertebrates (Heard and Martienssen 2014), where silent and active alleles of a given gene may coexist and segregate in a Mendelian manner during meiosis (Bošković and Rando 2018). Up to date, only one clear example exists in vertebrates of such epialleles, namely the agouti (*A*) gene model in mice, where different phenotypes produce offspring of similar coat colour without the presence of genetic variation for coat colour (Daxinger and Whitelaw 2012). However, since we here lack data from the P until the fourth generation, we were unable to actually track CpG methylation throughout the selection process.

Alternative reasons for the lack of a correlated response of CpG variation to artificial selection on exploratory behavior, might be because we have conducted only four generations of artificial selected for exploratory behavior. These four generations might not have been enough to fully fixate methylation differences between the lines associated with exploratory behavior. However, since we were able to observe ample genetic diversity between individuals after four generations (Laine et al. unpublished data), we assume that any co-selection on transgenerational inherited methylation would also be detectable. In an analysis on methylation differences between two red junglefowl lines, selected for divergent levels of fear for humans over five generations, several differences were found in hypothalamic DNA methylation between the lines (Belteky et al. 2018). Two differences in appraoch between the studies could explain the dissimilar findings with ours. First, instead of a CpG-based analysis, methylated DNA immunoprecipitation was used, analyzing 1-kb regions on global methylation differences. Second, where we have sequenced erythrocyte methylation, where genetic differences might be more apparent in other tissues such as brain tissue. In Great Tits, we have found in an earlier study that methylation differences between tissues are especially apperent for genes that are expressed in a tissue-specific way (Derks et al. 2016). So, where induced levels of DNA methylation in blood maybe a good biomarker of induced levels in other tissues, stably inherited methylation marks might be tissue specific. In our study on methylation differences in DRD4, however, we found a difference between the lines both in blood as well as in brain tissue (Verhulst et al. 2016).

Alternatively, there are several reasons why we could have missed potential CpGs. First of all, we have conducted our study on only a limited number of samples and libraries. By using pooled samples for WGBS we, on one hand, dilute any between family variation in the SEL samples and even corrected for that in the F2 inter-cross samples, on the other hand, we might not pick up any subtle differences that are only present in some of these families. We also show that by taking a WGBS approach, likely many CpG sites are taken into consideration that may not be relevant for this analysis, such as intergenic CpGs, although they could be relevant for transposable element methylation of CpG island methylation in other contexts (Derks et al. 2016). Another reason is that due to our strict correction for the number of tests we conducted, type II errors can be expected (failure to recognize differentially methylated sites as significant). Since we expected an association between the differences in methylation between the two SEL libraries and the two F2C libraries, we explored this correlation. We only found one CpG site that was consistently close to significance in both the SEL analyses and the F2 intercross WGBS. This indicates that if we missed any sites in our WGBS analysis on the SELs, they are likely not present in the F2 intercrosses and therefore likely caused by drift or founder effects. However, future analyses including individual-based data should give more power to detect potential sites. Another reason could be that we could have lower epigenetic variation in our captive population compared to a wild population. A meta-analysis showed, however, that genetic variation in personality traits is not higher under controlled lab experiments (*h*^2^ = 0.24) compared to wild populations (*h*^2^ = 0.36) (van Oers and Sinn 2013).

We did find seven differentially methylated sites in the RRBS analysis on the fast and slow F2 intercross individuals, and we were able to annotate six out of these seven DMC in genes. We found a DMC near the transcription termination site of one of the genes, *MRM1*, which is present in mitochondrial RNA granules and responsible for methylation of 16S mt-rRNA (Van Haute et al. 2015). Two other CpG’s were present in promotor regions of genes. The first is an interesting candidate to explain phenotypic differences in exploratory behavior and was identified as the scale keratin-like gene, a bird- and reptile-specific gene responsible for the structural constituent of the cytoskeleton. For example, in a genomic comparison between African and European chicken (*Gallus gallus*), a group of neghboring scale keratine-like genes was found to be under strong selection in relation to environmentally induced stress tolerance (Fleming et al. 2017), a likely component of exploratory behavior (Baugh et al. 2017). A second DMC in a promotor region was found in *SBNO2*. Three other significant differentially methylated sites were found in gene bodies. Two of which were in close proximity in semaphorin 7 A (John Milton Hagen blood group; *SEMA7A*) and one in the LPS Responsive Beige-Like Anchor gene* (LRBA)*. Although no CpGs were significantly differentially methylated in our WGBS datasets, one CpG reached significance in both the SEL comparison as well as the comparison between the SE and FE pool of the F2 inter-crosses. This CpG was situated near the transcription termination site of RNA polymerase II subunit C (*POLR2C*), a gene related to RNA transcription and RNA splicing. There are two reasons why these sites were not differentially methylated in the WGBS dataset of the selected lines. First, because we had more power in detecting significant CpGs in the RRBS dataset and second, since the phenotypic variation in EEB between the siblings of the F2 intercross individuals is likely not only due to heritable variation alone, these CpGs are indicative for functional variants that affect nonheritable phenotypic variation in exploratory behavior via environmental effects during early development. Therefore, the detected genes remain largely indicative and need further functional validation in the future.

In conclusion, we did not find evidence for a correlated selection on CpG methylation in lines selected for exploratory behavior in Great Tits. We did find that CpG methylation may explain phenotypic variation in exploratory behavior indicating that DNA methylation may be more important for plastic responses to the environment, rather than to be stably inherited over multiple generations. We infer from these results that there is low evolutionary potential for variation in DNA methylation related to exploratory behavior (Verhoeven et al. 2016; Herrel et al. 2020). We do, however, confirm that variation in DNA methylation affects the noninherited fraction of behavioral phenotypes, which is now been recognized in many systems (Yan et al. 2015; Merlin and Liedvogel 2019; Sepers et al. 2019; Lindner et al. 2020). From this, we conclude that DNA methylation mainly plays a role for epigenetically based phenotypic plasticity, and that these environmentally induced effects of DNA methylation on behavioral traits in vertebrates will likely not play a role in the evolution of such traits (Heard and Martienssen 2014).

## Supplementary Material

icaa138_supplementary_data

## Notes

From the symposium “Epigenetic Variation in Endocrine Systems” presented at the annual meeting of the Society for Integrative and Comparative Biology, January 3–7, 2020 at Austin, Texas.
