# Supplementary material for: Epigenetics of Animal Personality: DNA Methylation Cannot Explain the Heritability of Exploratory Behavior in a Songbird
Source: Integr Comp Biol. 2020 Nov 12;60(6):1517–30. doi: 10.1093/icb/icaa138 (PMC7742756; doi:10.1093/icb/icaa138)

Supplementary Table 1. Sample information and WGBS sequencing and mapping information.

|  | # Total reads | Duplication (%) | GC (%) | Mapping efficiency (%) | # CpG sites before filter | # CpG sites after 10x filter | # CpG sites after 10x and % filter | # CpG sites in both samples |
| --- | --- | --- | --- | --- | --- | --- | --- | --- |
| FE-SEL | 205,312,498 | 33.09 | 27 | 66.02 | 14,425,279 | 3,139,682 | 2,376,733 | 2,023,034 |
| SE-SEL | 323,619,782 | 22.57 | 27 | 64.43 | 14,771,423 | 8,737,120 | 6,148,189 |  |
| FE-F2C | 334,713,620 | 23.49 | 28 | 57.38 | 15,188,071 | 11,526,013 | 8,458,226 | 6,451,826 |
| SE-F2C | 287,425,864 | 22.57 | 28 | 51.14 | 15,153,795 | 10,708,052 | 8,217,676 |  |

Supplementary Table 2. Sample information and RRBS sequencing and mapping information.

|  | EEB Type | EEB Score | Family nr | Lane nr | # Total reads | Duplication (%) | GC (%) | Mapping efficiency (%) | # CpG sites before filter | # CpG sites after 10x filter | # CpG sites after 10x and % filter |
| --- | --- | --- | --- | --- | --- | --- | --- | --- | --- | --- | --- |
| AB.77958 | FE | 16 | 1 | 2 | 18,910,812 | 75.84 | 37 | 65.65 | 3,864,311 | 2,588,539 | 1,047,382 |
| AB.77959 | SE | 5 | 1 | 1 | 16,325,088 | 73.72 | 36 | 66.57 | 3,909,059 | 2,346,873 | 948,512 |
| AB.77970 | SE | 0 | 2 | 2 | 17,685,553 | 74.79 | 37 | 66.05 | 3,700,483 | 2,545,205 | 1,038,160 |
| AB.77974 | SE | 15 | 2 | 1 | 16,906,367 | 74.39 | 37 | 66.42 | 3,753,646 | 2,426,688 | 983,656 |
| AF.08933 | SE | 0 | 3 | 1 | 16,585,586 | 76.25 | 38 | 65.44 | 3,706,608 | 2,167,437 | 854,906 |
| AF.08974 | FE | 15 | 3 | 2 | 19,622,050 | 71.31 | 36 | 66.14 | 4,157,643 | 2,603,890 | 1,055,027 |
| AR.04022 | FE | 14 | 4 | 2 | 17,321,465 | 74.82 | 36 | 67.93 | 3,750,778 | 2,614,435 | 1,048,989 |
| AR.04024 | SE | 0 | 4 | 1 | 18,452,793 | 77.06 | 38 | 66.02 | 3,790,862 | 2,460,250 | 986,686 |
| AR.04065 | SE | 0 | 5 | 2 | 18,706,590 | 75.61 | 37 | 65.61 | 3,746,709 | 2,615,373 | 1,102,466 |
| AR.04070 | FE | 16 | 5 | 1 | 17,002,131 | 76.24 | 38 | 65.57 | 3,793,894 | 2,369,285 | 932,340 |
| AR.04081 | FE | 15 | 6 | 1 | 17,564,386 | 75.77 | 37 | 65.66 | 3,683,308 | 2,482,856 | 1,014,162 |
| AR.04194 | SE | 0 | 6 | 2 | 18,827,347 | 75.53 | 37 | 65.77 | 3,810,698 | 2,617,761 | 1,072,192 |
| F.858680 | SE | 0 | 7 | 1 | 19,518,932 | 77.53 | 38 | 65.51 | 3,820,739 | 2,504,313 | 994,575 |
| F.858683 | FE | 17 | 7 | 2 | 17,340,207 | 74.83 | 37 | 65.32 | 3,739,402 | 2,463,808 | 985,004 |
| F.858920 | SE | 0 | 8 | 2 | 18,575,257 | 74.79 | 37 | 65.80 | 3,908,824 | 2,553,498 | 1,070,835 |
| F.858924 | FE | 14 | 8 | 1 | 17,773,578 | 75.63 | 37 | 64.85 | 3,776,454 | 2,303,298 | 946,725 |

Supplementary Figure 1.

Quality of the reads for the four WGBS libraries a) before trimming and b) after trimming.

a.


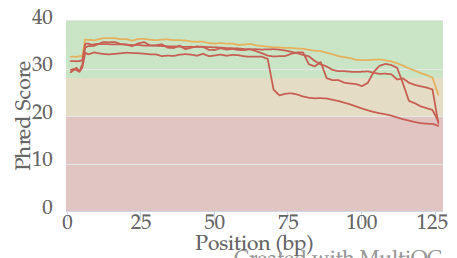


b.


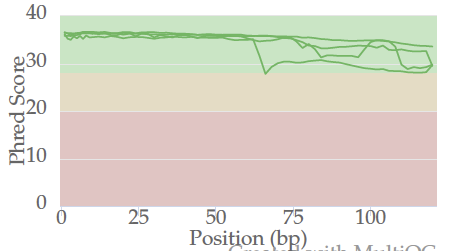


Supplementary Figure 2.

Dendrogram showing how the F2C individuals are clustered based on the similarity of their methylation profiles. Individual names are given with F (fast; blue) or S (slow; red) indicating their phenotype, a number from 1 to 7 indicating their family and their ring number.


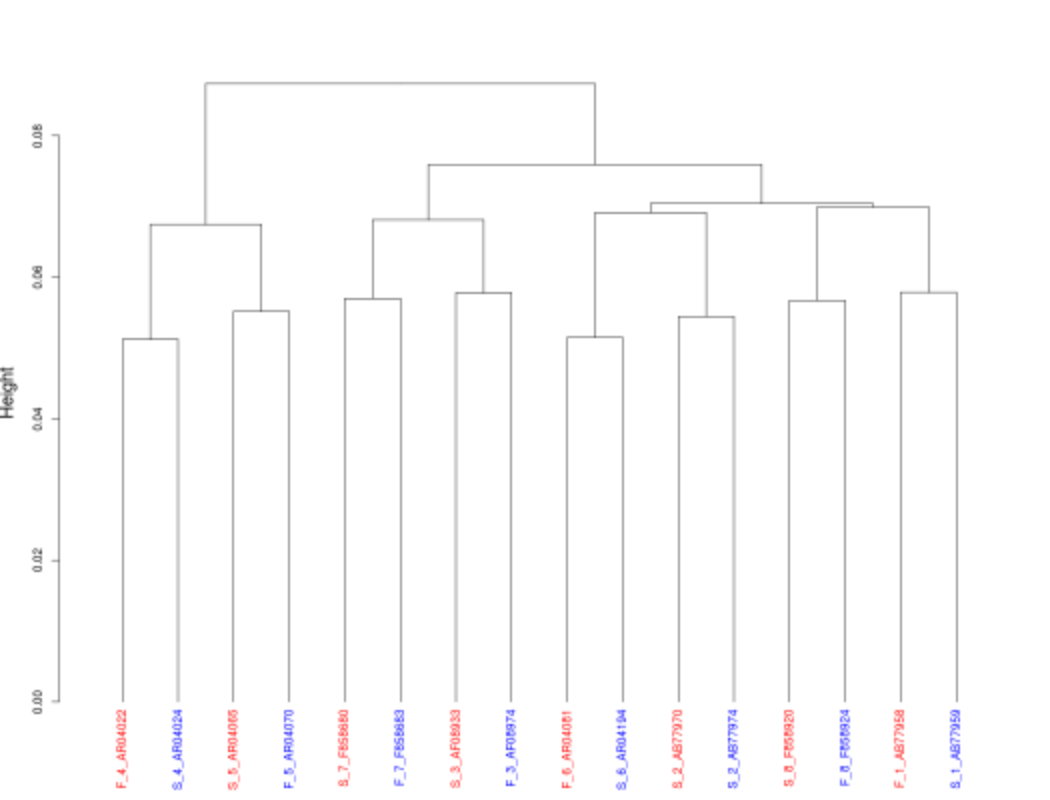


Supplementary Figure 3.

PCA plot of F2C individuals based on the PCA analysis of their methylation profiles showing PC1 against PC2.


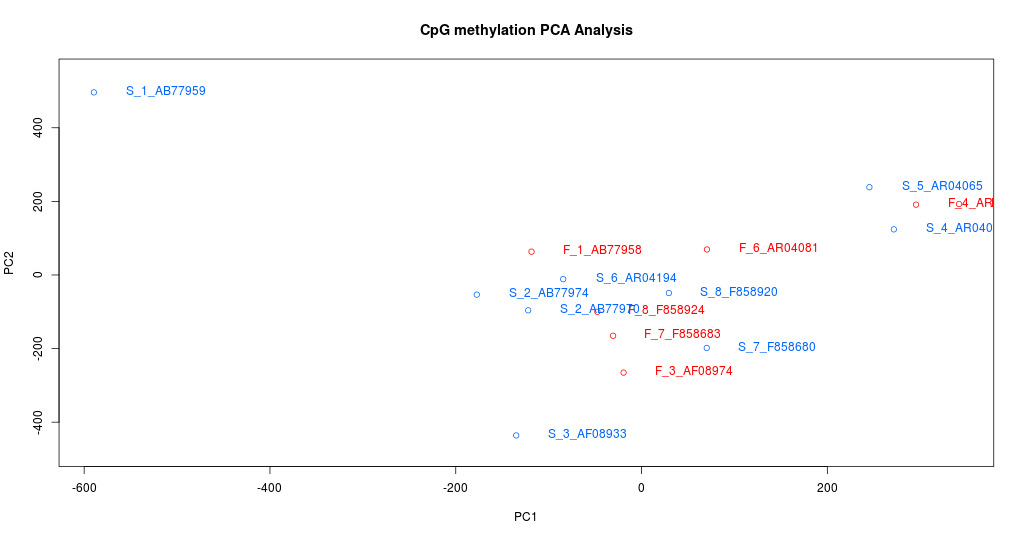


Supplementary Figure 4.

QQplot depicting the expected -log(p) values against the observed -log(p) values for the WGBS analysis for a) the pooled selection lines (SEL), b) the pooled F2 cross families (F2C-FAM) and c) the RRBS analysis on the individual samples of the F2 cross (F2C-IND).


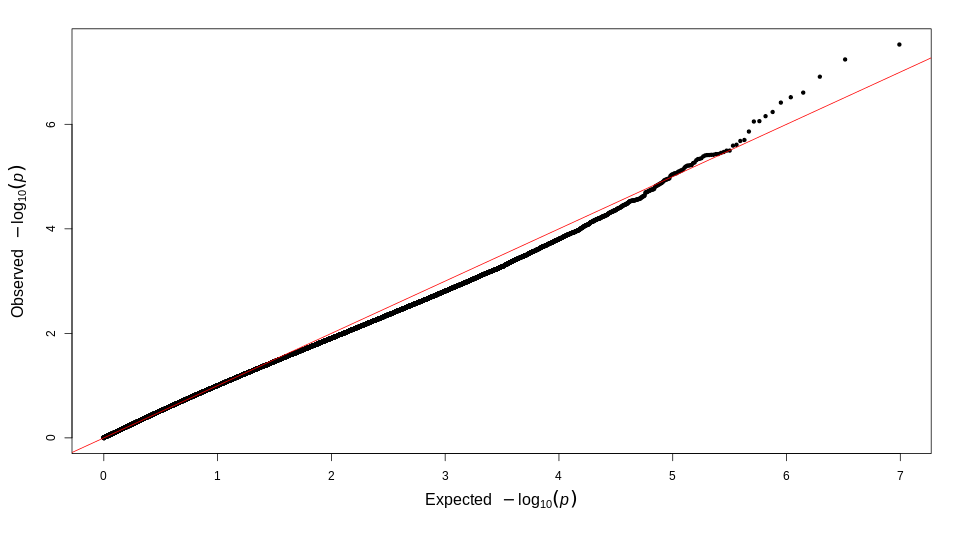

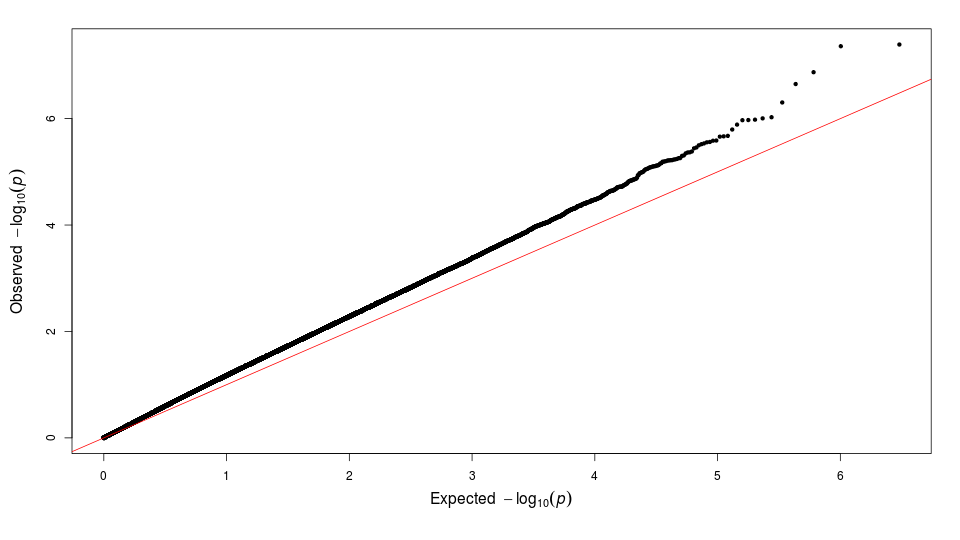


a.

b.


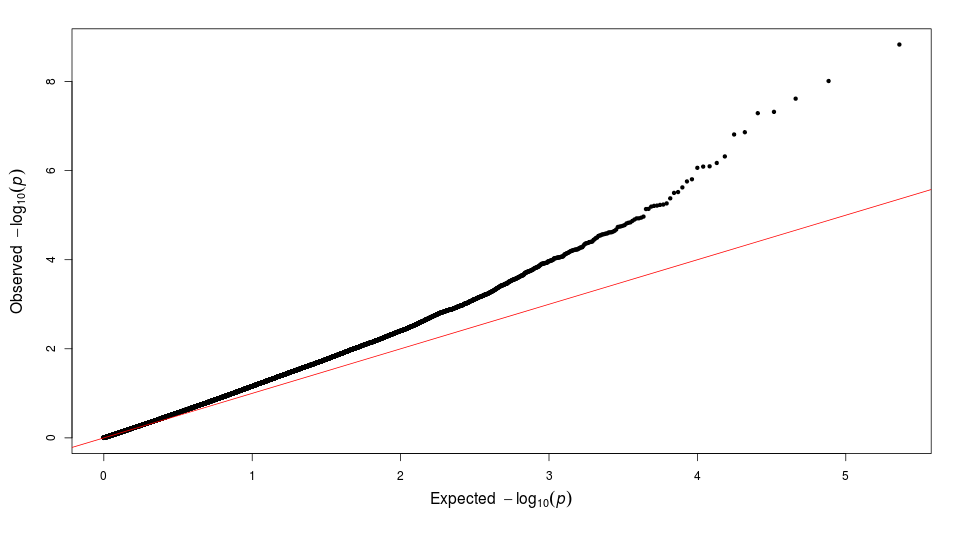


c.

Supplementary Figure 5.

Overdispersion statistics before and after filtering of the GLMM for RRBS F2C-IND. The 95% Highest Posterior Density, is indicated by the blue arrow was used and the overdispersed sites were filtered out from further analyses.


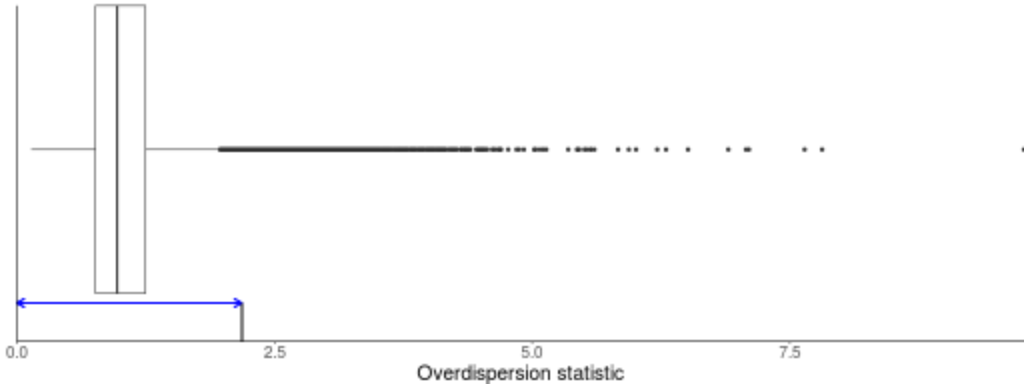


Supplementary Figure 6.

CpG site coverage against the significance from the GLMM for RRBS F2C-IND. Each plot represents one individual.


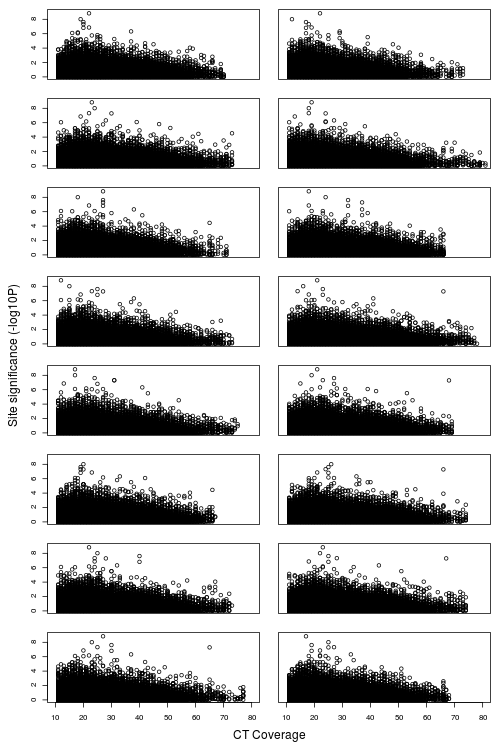


Supplementary Figure 7.

Significance values (-log_10_(P)) derived from the differential methylation analysis for a) the significance values (-log_10_(P)) derived from the differential methylation analysis for the WGBS SEL libraries (FE – SE) against the significance values (-log_10_(P)) derived from the differential methylation analysis for the WGBS F2C individuals (FE – SE) for 1,382,302 CpGs that were present in both analyses. In b) the WGBS F2C libraries (FE – SE) against the significance values (-log_10_(P)) derived from the differential methylation analysis for the RRBS F2C individuals (FE – SE) for 80,300 CpGs that were present in both analyses are given.


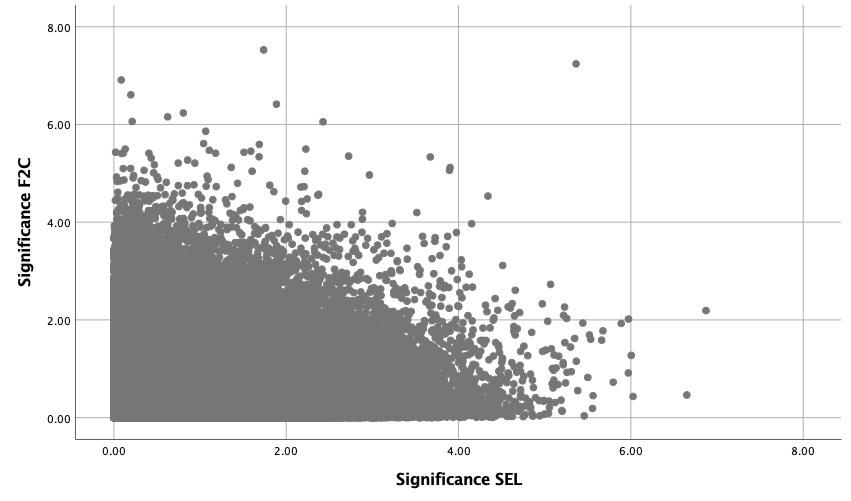


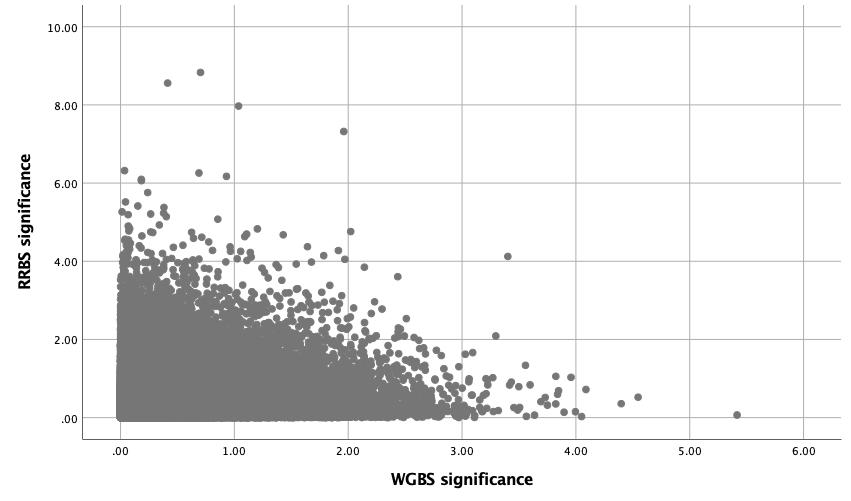

Supplement: icaa138_supplementary_data [file icaa138_supplementary_data.docx]
